# Prevalence of nonalcoholic fatty liver disease in mental disorder inpatients in China: an observational study

**DOI:** 10.1007/s12072-020-10132-z

**Published:** 2021-01-29

**Authors:** Qiuyue Ma, Fude Yang, Botao Ma, Wenzhan Jing, Jue Liu, Moning Guo, Juan Li, Zhiren Wang, Min Liu

**Affiliations:** 1 Department of Epidemiology and Biostatistics, School of Public Health, Peking University, No.38, Xueyuan Road, Haidian District, Beijing, 100191 China; 2 Beijing Huilongguan Hospital, Peking University Huilonguan Clinical Medical School, No.7, Huilongguan Nandian Road, Changping District, Beijing, 100096 China; 3 Beijing Municipal Commission of Health and Family Planning Information Center, Beijing Municipal Commission of Health and Family Planning Policy Research Center, No.277, Zhaodengyu Road, Xicheng District, Beijing, 100034 China; 4 Beijing Geriatric Hospital, No.118, Wenquan Road, Haidian District, Beijing, 100095 China

**Keywords:** Nonalcoholic fatty liver disease, Mental disorder, Schizophrenia, Bipolar disorder, Depressive disorder, Prevalence, Chronic liver disease, NAFLD, Liver steatosis, China

## Abstract

**Background and Purpose:**

Non-alcoholic fatty liver disease (NAFLD) is becoming the most common liver disease in China. However, the understanding of NAFLD prevalence among Chinese mental disorder inpatients remains insufficient. We aim to investigate the prevalence of NAFLD among mental disorder inpatients in Beijing, China.

**Methods:**

In this observational study, we included 66,273 mental disorder inpatients between 2014 and 2018, including inpatients with schizophrenia, bipolar disorder, depressive disorder and other mental disorders. Data were obtained from electronic health records of 19 specialized psychiatric hospitals in Beijing. NAFLD was defined by ICD-10 code, excluding other causes of liver disease. We calculated the overall and annual prevalence rates of NAFLD from 2014 to 2018, and compared NAFLD prevalence between sexes, age groups, mental disorders types, antipsychotics use, and comorbidities. Multivariable logistic regression was used to examine risk factors associated with NAFLD. Subgroup analysis was performed in different mental disorder types.

**Results:**

The prevalence of NAFLD was 17.63% (95% CI 17.34–17.92%) in mental disorder inpatients, increasing from 16.88% in 2014 to 19.07% in 2018. The NAFLD prevalence in participants with schizophrenia (22.44%) was higher than that in participants with bipolar disorder (17.89%), depressive disorder (12.62%), and other mental disorders (12.99%). Women had similar or even higher NAFLD prevalence than men after 50 years. Men, 50–59 years (aOR = 1.71), schizophrenia (aOR = 1.56), bipolar disorder (aOR = 1.47), antipsychotics use (aOR = 1.46), hypertension (aOR = 1.50), diabetes (aOR = 1.83), dyslipidemia (aOR = 2.50) were risk factors for NAFLD in mental disorder inpatients.

**Conclusion:**

NAFLD was common among Chinese mental disorder inpatients, and increased over years. The prevalence of NAFLD was higher among men, old women, inpatients with schizophrenia, bipolar disorder, antipsychotics, hypertension, diabetes, and dyslipidemia. Fatty liver disease among mental disorder population warrants the attention of psychiatric specialists and health policy-makers.

**Supplementary Information:**

The online version contains supplementary material available at 10.1007/s12072-020-10132-z.

## Introduction

Non-alcoholic fatty liver disease (NAFLD) is the most common liver disease in the world [1]. It is a major cause of cirrhosis and liver cancer, which cause significant morbidity and mortality [1, 2]. During the last two decades, the prevalence of other chronic liver diseases has remained stable or decreased, while the prevalence of NAFLD has doubled. [3, 4] The estimated global prevalence of NAFLD is 25.24%, with the highest prevalence in South America (31%) and the Middle East (32%) and the lowest in Africa (14%) [5]. In China, the prevalence of NAFLD in adults was estimated to be 20.1–29.2%, and has increased over time [6, 7]. NAFLD is closely associated with metabolic syndrome, obesity, type 2 diabetes, and dyslipidemia [8–10]. As the epidemics of obesity and type 2 diabetes increase worldwide, NAFLD is increasing proportionately, and has become a major public health issue worldwide [11, 12].

Evidences showed that the risk of metabolic syndrome was elevated in mental disorder patients, such as patients with schizophrenia, bipolar disorder, and major depressive disorder [13, 14]. Obesity, hypertension, dyslipidemia, and type 2 diabetes are more common in people with mental disorders, partly due to adverse health behaviors, such as low levels of physical activity and poor diet [15]. As NAFLD was considered as the hepatic independent manifestation of the metabolic syndrome, [16] it is expected that NAFLD will be more common among people with mental disorders. For example, patients with schizophrenia had higher prevalence of chronic liver disease than the general population, due to higher prevalence of metabolic syndrome, unhealthy lives, and antipsychotics use [17]. Besides, patients with bipolar disorder were reported to have higher prevalence of liver diseases [18]. A recent study conducted in a nationally representative sample of US population showed that depression was independently associated with NAFLD [19].

Despite tremendous research in NAFLD among general population, the understanding of NAFLD prevalence among Chinese mental disorder inpatients remains insufficient. Compared to western population, the lifestyle and genetic factors causing NAFLD were different in Chinese population [20]. A recent cross-sectional study found that the prevalence of NAFLD was 49.5% in young males with schizophrenia, [21] which highlighted the need for more attention to fatty liver disease in schizophrenia patients. However, this above study only included 202 cases of young male schizophrenia patients, without female and elder participants [21]. Furthermore, the prevalence of NAFLD has not been clearly described in patients with bipolar disorder and depressive disorder. To our knowledge, no large study has been performed to investigate the prevalence and risk factors of NAFLD among mental disorder inpatients in China.

In this observational study, we aim to investigate the prevalence and trend of NAFLD among mental disorder inpatients in Beijing, China, and analyze the risk factors for NAFLD among mental disorder inpatients. We aim to bring more attention to fatty liver disease in mental disorder inpatients, and reduce the burden of physical disease in people with mental disorders.

## Methods

### Study design and participants

This observational study included adult mental disorder inpatients in 19 specialized psychiatric hospitals in Beijing between January 1, 2014 and December 31, 2018. This study was based on data from electronic health records provided by Beijing Municipal Commission of Health and Family Planning Information Center, which compiled information of demographic characteristics, discharge diagnoses, and treatment procedures obtained directly and in anonymous format from hospital information systems. Among the 19 hospitals, four are third-level hospitals, which have relatively more medical resources, and 15 are second-level hospitals, which are regional medical centers. Mental disorders and physical diseases were diagnosed according to the 10th revision of the International Classification of Diseases (ICD-10) [22].

Inclusion criteria for this study were mental disorder inpatients aged at least 18 years who were after hospitalized in the abovementioned 19 hospitals between 2014 and 2018 (88,535). Exclusion criteria were inpatients who were re-admitted within the same calendar year (19,463), and those who were diagnosed as mental and behavioral disorder due to use of alcohol (2799). A total of 66,273 inpatients were included in the final analysis, including inpatients with schizophrenia (ICD-10 code: F20), bipolar disorder (F31), depressive disorder (F32 and F33) and other mental disorders.

### Definition of NAFLD

The definition of NAFLD requires evidence of hepatic steatosis by either imaging or histology and absence of other causes of hepatic fat accumulation from conditions, such as significant alcohol consumption, hepatitis C, medication use, or hereditary disorders [23]. Liver ultrasounds were performed by experienced medical workers, and NAFLD was diagnosed by physicians based on the ultrasound examinations and disease history. In this study, ICD-10 code for NAFLD is K76.0. After identification of inpatients with ICD-10 code of K76.0, we further excluded those with alcoholic liver disease (K70), toxic liver disease (K71), viral hepatitis (B15-B19), autoimmune hepatitis (K75.4), biliary cirrhosis (K74.3-K74.5), hemochromatosis (E83.1), and Wilson's disease (E83.0).

### Comorbidity

Comorbidities associated with NAFLD were identified by ICD-10 codes, including hypertension (I10-I15), diabetes (E10-E14), and dyslipidemia (E78).

### Covariates

Covariates that may be associated with NAFLD in this study included demographic characteristics (sex, age, ethnic origin, marital status, payment, hospital level), mental disorder factors (mental disorder types, antipsychotics use), and comorbidities (hypertension, diabetes, dyslipidemia). Age was classified into six categories: 18–29, 30–39, 40–49, 50–59, 60–69, and ≥ 70 years. Ethnic origin was divided into Han ethnic and other ethnic. Marital status was classified into three categories: never married, married, divorced or widowed. Payment was classified into five categories: new rural cooperative medical scheme (NCMS), urban employee basic medical insurance (UEBMI), urban resident basic medical insurance (URBMI), other insurance, and out-of-pocket (OOP) expenditure. Hospital level was classified into third level and second level. Mental disorder was divided into four categories: schizophrenia, bipolar disorder, depressive disorder, and other mental disorders. Antipsychotics use, hypertension, diabetes, dyslipidemia were divided into two categories (no, yes), respectively.

### Statistical analysis

Demographic characteristics, mental disorder factors, and comorbidities were described by proportion. The prevalence of NAFLD was reported as percentages and 95% confidence intervals (CIs). Annual prevalence rates of NAFLD from 2014 to 2018 were calculated for all participants and separately for men and women. Age-adjusted prevalence was calculated using direct standardization method, with all participants as reference population. We used *χ*^2^ test to compare crude NAFLD prevalence rates between different groups. Multivariable logistic regression was used to examine the association of all covariates with the prevalence of NAFLD. Adjusted odds ratios (aORs) and 95% CIs were calculated.

Considering that the prevalence of NAFLD varies in different mental disorders, we did several subgroup analyses for different mental disorder types. The age-specific prevalence of NAFLD by sex was calculated separately for schizophrenia, bipolar disorder, depressive disorder. The prevalence of NAFLD in patients with comorbidities was also calculated separately for different mental disorder types. We also performed multivariable logistic regressions for different mental disorder types. All p values were 2-tailed and *p* < 0.05 was the threshold for statistical significance. All statistical analyses were performed with R version 4.0.2 (R Foundation).

## Results

A total of 66,273 mental disorder inpatients were included in this study. The basic characteristics of participants were as follows: 35,586 (53.70%) were women, 30,253 (45.65%) were aged 18–39 years, 62,935 (94.96%) were Han ethnic, 31,964 (48.23%) were married, 27,532 (41.54%) had UEBMI, 55,392 (83.58%) were from third-level hospitals. 25,503 (38.48%) had schizophrenia, 14,377 (21.69%) had bipolar disorder, 11,406 (17.21%) had depressive disorder, 14,987 (22.61%) had other mental disorders. Of all participants, 49,210 (74.25%) inpatients used antipsychotics. 12,127 (18.30%) had hypertension, 8559 (12.91%) had diabetes, 12,568 (18.96%) had dyslipidemia (Table 1).

**Table 1:** Prevalence of NAFLD in mental disorder inpatients in 2014–2018, Beijing, China

	Number (%)	NAFLD (*n*)	Prevalence (%(95% CI))	*P* value	aOR (95% CI)
Total	66,273 (100.00)	11,681	17.63 (17.34–17.92)		
Sex				< 0.001	
Men	30,687 (46.30)	5814	18.95 (18.51–19.39)		1.00
Women	35,586 (53.70)	5867	16.49 (16.10–16.88)		0.88 (0.84–0.92)
Age group (years)				< 0.001	
18–29	18,376 (27.73)	1825	9.93 (9.50–10.37)		1.00
30–39	11,877 (17.92)	2022	17.02 (16.35–17.71)		1.49 (1.38–1.60)
40–49	10,262 (15.48)	2018	19.66 (18.90–20.45)		1.52 (1.40–1.65)
50–59	11,592 (17.49)	2858	24.65 (23.87–25.45)		1.71 (1.58–1.86)
60–69	8393 (12.66)	1990	23.71 (22.80–24.64)		1.39 (1.27–1.52)
≥ 70	5773 (8.71)	968	16.77 (15.81–17.76)		0.89 (0.79–0.99)
Ethnic origin				< 0.001	
Han	62,935 (94.96)	11,192	17.78 (17.49–18.08)		1.00
Other	3338 (5.04)	489	14.65 (13.47–15.89)		0.89 (0.80–0.99)
Marriage				< 0.001	
Never married	26,056 (39.32)	3953	15.17 (14.74–15.61)		1.00
Married	31,964 (48.23)	5772	18.06 (17.64–18.48)		1.03 (0.97–1.09)
Divorced/widowed	8253 (12.45)	1956	23.70 (22.79–24.63)		1.08 (1.00–1.16)
Payment				< 0.001	
NCMS	4750 (7.17)	784	16.51 (15.46–17.59)		1.00
UEBMI	27,532 (41.54)	6249	22.70 (22.20–23.20)		1.15 (1.06–1.26)
URBMI	4963 (7.49)	1162	23.41 (22.24–24.62)		1.16 (1.04–1.29)
Other insurance	12,574 (18.97)	1206	9.59 (9.08–10.12)		0.68 (0.61–0.75)
OOP	16,454 (24.83)	2280	13.86 (13.33–14.39)		0.95 (0.87–1.04)
Hospital level				< 0.001	
Third level	55,392 (83.58)	9127	16.48 (16.17–16.79)		1.00
Second level	10,881 (16.42)	2554	23.47 (22.68–24.28)		1.14 (1.07–1.21)
Mental disorder				< 0.001	
Other	14,987 (22.61)	1947	12.99 (12.46–13.54)		1.00
Schizophrenia	25,503 (38.48)	5723	22.44 (21.93–22.96)		1.56 (1.46–1.66)
Bipolar disorder	14,377 (21.69)	2572	17.89 (17.27–18.53)		1.47 (1.38–1.58)
Depressive disorder	11,406 (17.21)	1439	12.62 (12.01–13.24)		0.91 (0.84–0.99)
Antipsychotic**s**				< 0.001	
No	17,063 (25.75)	2302	13.49 (12.98–14.01)		1.00
Yes	49,210 (74.25)	9379	19.06 (18.71–19.41)		1.46 (1.38–1.54)
Hypertension				< 0.001	
No	54,146 (81.70)	8353	15.43 (15.12–15.73)		1.00
Yes	12,127 (18.30)	3328	27.44 (26.65–28.25)		1.50 (1.41–1.58)
Diabetes				< 0.001	
No	57,714 (87.09)	8784	15.22 (14.93–15.52)		1.00
Yes	8559 (12.91)	2897	33.85 (32.84–34.86)		1.83 (1.73–1.94)
Dyslipidemia				< 0.001	
No	53,705 (81.04)	7252	13.50 (13.22–13.80)		1.00
Yes	12,568(18.96)	4429	35.24 (34.40–36.08)		2.50 (2.38–2.62)

*NAFLD* Nonalcoholic fatty liver disease. *aOR* adjusted odds ratio. *NCMS* new rural cooperative medical scheme. *UEBMI* urban employee basic medical insurance. *URBMI* urban resident basic medical insurance. *OOP* out-of-pocket expenditure

**Fig. 1:**
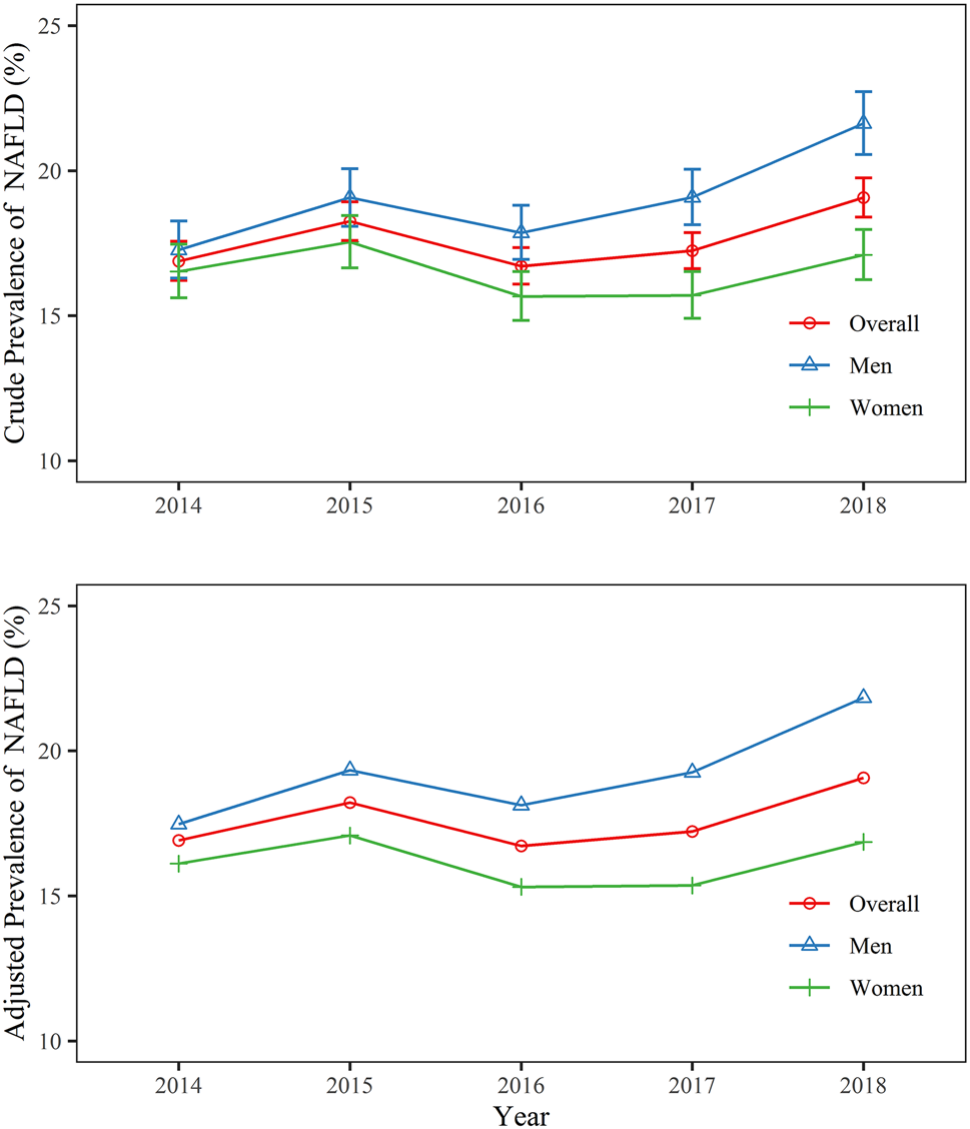
Crude and adjusted prevalence of NAFLD in mental disorder inpatients in 2014–2018, Beijing, China

Of the 66,273 participants, we identified 11,737 inpatients with ICD-code of K76.0. We further excluded inpatients with alcoholic liver disease (8), toxic liver disease (11), viral hepatitis (36), and autoimmune hepatitis (1). Finally, a total of 11,681 inpatients had NAFLD, the prevalence was 17.63% (95% CI 17.34–17.92%), with 18.95% (95% CI 18.51–19.39%) in men and 16.49% (95% CI 16.10–16.88%) in women. From 2014 to 2018, the prevalence of NAFLD increased from 16.88% (95% CI 16.22–17.57%) to 19.07% (95% CI 18.40–19.75%) (*p* for trend = 0.002). The overall standardized prevalence of NAFLD increased from 16.91% in 2014 to 19.07% in 2018. The standardized prevalence increased from 17.47 to 21.83% for men, and increased from 16.11 to 16.86% for women (Fig. 1).

The prevalence of NAFLD was higher in men (18.95%), 50–59 years group (24.65%), Han ethnic (17.78%), divorced or widowed (23.70%), participants with URBMI (23.41%), participants from second-level hospitals (23.47%). The NAFLD prevalence in participants with schizophrenia (22.44%) was higher than in participants with bipolar disorder (17.89%), depressive disorder (12.62%), and other mental disorders (12.99%). Participants who used antipsychotics had higher NAFLD prevalence (19.06%) than those who didn’t (13.49%). The prevalence was significantly higher in participants with hypertension (27.44%), diabetes (33.85%), and dyslipidemia (35.24%) than those without these comorbidities (Table 1).

In the multivariable logistic models for NAFLD, the aOR for women was 0.88 (95% CI 0.84–0.92); for 50–59 years the aOR was 1.71 (95% CI 1.58–1.86); for other ethnic the aOR was 0.89 (95% CI 0.80–0.99); for divorced or widowed participants the aOR was 1.08 (95% CI 1.00–1.16). The aOR was 1.15 (95% CI 1.06–1.26) for participants with UEBMI and 1.16 (95% CI 1.04–1.29) for participants with URBMI, the aOR for participants from second-level hospitals was 1.14 (95% CI 1.07–1.21).

Compared with other mental disorders, the aORs were 1.56 (95% CI 1.46–1.66) for schizophrenia, 1.47 (95% CI 1.38–1.58) for bipolar disorder, and 0.91 (95% CI 0.84–0.99) for depressive disorder. The aOR for antipsychotics use was 1.46 (95% CI 1.38–1.54), for hypertension was 1.50 (95% CI 1.41–1.58), for diabetes was 1.83 (95% CI 1.73–1.94), for dyslipidemia was 2.50 (95% CI 2.38–2.62, Table 1).

**Fig. 2:**
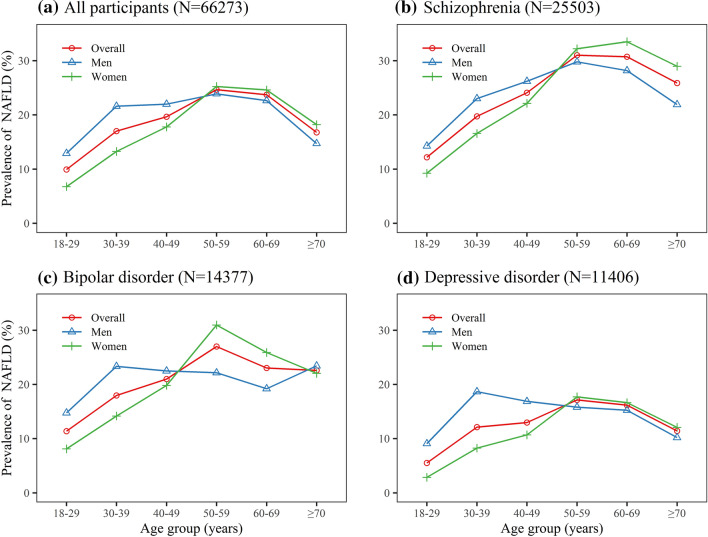
Age-specific prevalence of NAFLD among mental disorder inpatients, stratified by sex **a** Age-specific prevalence of NAFLD among all participants. **b** Age-specific prevalence of NAFLD among schizophrenia inpatients. **c** Age-specific prevalence of NAFLD among bipolar disorder inpatients. **d** Age-specific prevalence of NAFLD among depressive disorder inpatients

We calculated the age-specific prevalence of NAFLD for different mental disorder types after stratifying participants according to sex. Regardless of mental disorder types, men had higher NAFLD prevalence than women before 50 years. For participants aged more than 50 years, women had similar or even higher NAFLD prevalence than men. In schizophrenia inpatients, the prevalence of NAFLD in women peaked at 60–69 years (33.50%), while the prevalence in men peaked at 50–59 years (29.78%). In bipolar disorder inpatients, the prevalence of NAFLD in women peaked at 50–59 years (30.96%), while the prevalence in men peaked at 30–39 years (23.33%) and more than 70 years (23.42%). In depressive inpatients, the prevalence of NAFLD in women peaked at 50–59 years (17.71%), while the prevalence in men peaked at 30–39 years (18.66%, Fig. 2).

**Fig. 3:**
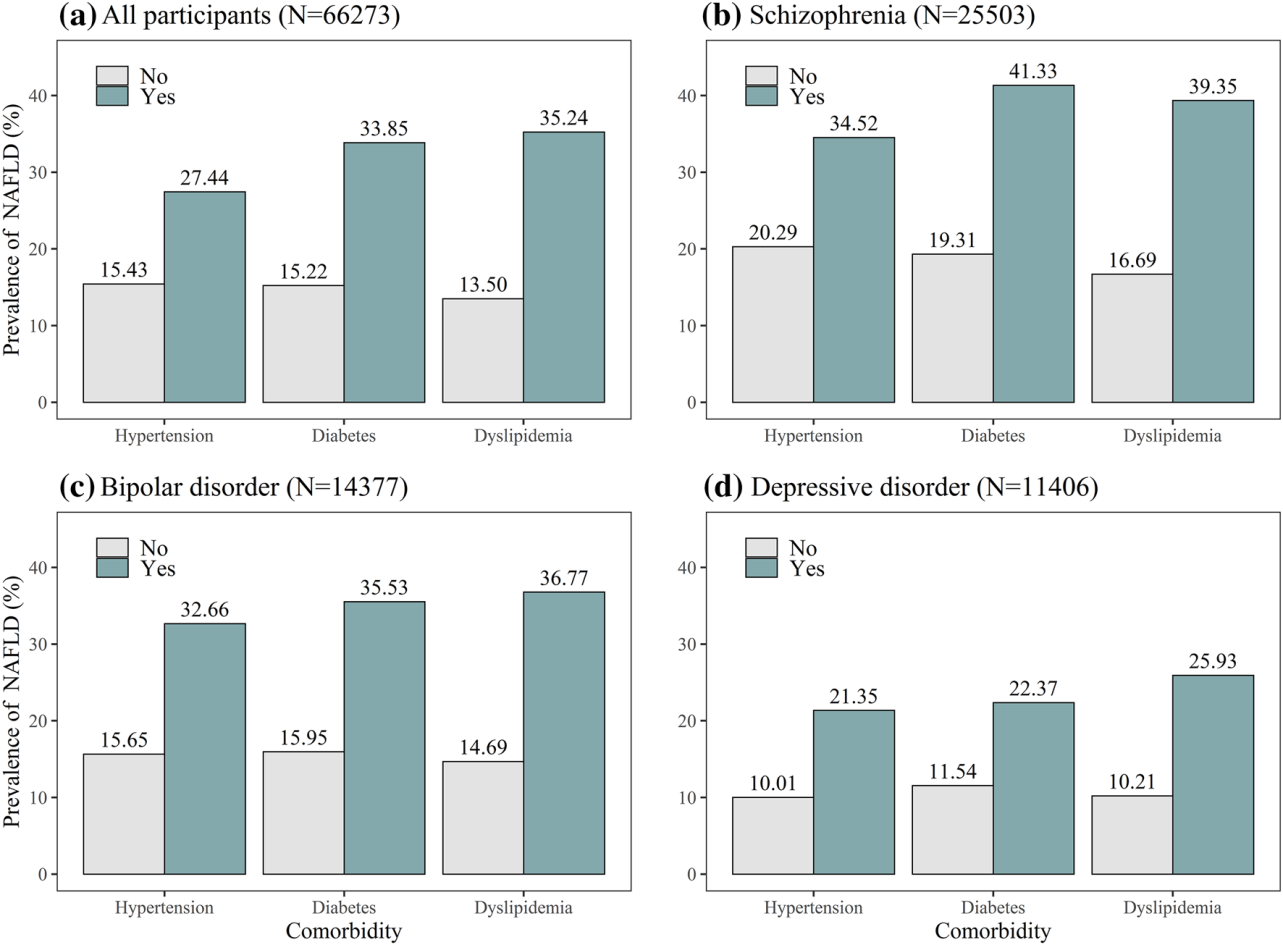
Prevalence of NAFLD among mental disorder inpatients with different comorbidities **a** Prevalence of NAFLD among all participants with different comorbidities. **b** Prevalence of NAFLD among schizophrenia inpatients with different comorbidities. **c** Prevalence of NAFLD among bipolar disorder inpatients with different comorbidities. **d** Prevalence of NAFLD among depressive disorder inpatients with different comorbidities

Regardless of mental disorder types, participant with hypertension, diabetes, dyslipidemia had higher prevalence of NAFLD than participants without these comorbidities. In schizophrenia inpatients, those with diabetes had the highest NAFLD prevalence (41.33%). In bipolar disorder and depressive disorder inpatients, those with dyslipidemia had the highest NAFLD prevalence (36.77 and 25.93%, respectively). Regardless of comorbidities, the prevalence of NAFLD was higher in participants with schizophrenia than in those with bipolar disorder and depressive disorder (Fig. 3).

**Fig. 4:**
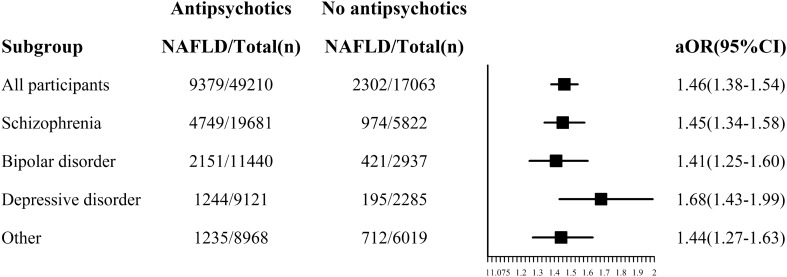
Association between antipsychotics and NAFLD by mental disorder types Adjusted for sex, age groups, ethnic origin, marriage, payment, hospital level, hypertension, diabetes, dyslipidemia

After adjusting for all the covariates, results of multivariable logistic regression showed that antipsychotics were associated with higher prevalence of NAFLD, regardless of mental disorder type. In addition, the aOR (1.68, 95% CI 1.43–1.99) was higher among inpatients with depressive disorder than among other types of mental disorder (Fig. 4).

## Discussion

NAFLD is a spectrum of diseases that include simple steatosis, nonalcoholic steatohepatitis (NASH), fibrosis, cirrhosis, and its complications [8, 24]. Since simple steatosis is often benign and reversible, early detection and intervention can help manage the growth of NAFLD among mental disorder inpatients [10, 20]. In a systematic review study, the prevalence of fatty liver disease in China was predicted to reach 20.21% in 2020, increasing at a rate of 0.594% per year [25]. In our study, it was estimated that 17.63% of mental disorder inpatients had NAFLD. Between 2014 and 2018, the prevalence of NAFLD increased from 16.88 to 19.07%, increasing at a rate of 0.548% per year. The increasing trend was similar to the growth of fatty liver disease in Chinese general population [25]. Consistent with the general population, urbanization, Westernized lifestyle, along with obesity and diabetes epidemic may partly explain the growing NAFLD prevalence among mental disorder inpatients [25]. Other important reason for increasing NAFLD prevalence included the adverse health behaviors, elevated metabolic syndrome risk, and antipsychotics use in mental disorder inpatients [13, 15]. Thus, public health campaigns are needed to increase awareness and diagnosis for NAFLD among mental disorder inpatients [20].

In this study, we found that the prevalence of NAFLD among mental disorder inpatients increased during 18–59 years, then started declining after 60 years, which showing an inverted *U*-shaped curve. This finding was consistent with studies in general Chinese population [6]. A meta-analysis showed that the pooled prevalence of NAFLD increased over age, peaked at 60 years, and decreased for people more than 60 years [6]. Given that risk factors for fatty liver disease development tend to increase with advancing age, middle aged and elderly are more likely to have NAFLD [11, 26]. Meanwhile, the decreased prevalence of NAFLD after 60 years may be the result of survivorship bias, as NAFLD was related to an increased morbidity and mortality of cardiovascular disease [6].

The prevalence of NAFLD was significantly higher in men than in women. During the reproductive age (18–49 years), women had lower NAFLD prevalence than men. However, women had a similar or even higher NAFLD prevalence after 50 years, compared to men of the same age. This finding was consistent with the notion that NAFLD occurred at a higher rate in women after menopause [27]. In women of reproductive age, the prevalence of NAFLD is lower than in men owing to the protective effect of estrogens, which wanes after the menopause [28]. Furthermore, postmenopausal women are more susceptible to weight gain, fat redistribution, and dyslipidemia, all major risk factors associated with NAFLD [29]. Thus, men and old women after menopause were high-risk population for NAFLD. Effective prevention measures focusing on these high-risk populations will have a profound impact on public health.

We found that the prevalence of NAFLD was associated with mental disorder types. In our study, the prevalence of NAFLD among inpatients with schizophrenia was 22.44%, higher than bipolar disorder and depressive disorder. A study in veterans showed the prevalence of overall liver disease in veterans with schizophrenia was 22.4%, [18] which was similar to the NAFLD prevalence among schizophrenia inpatients in our study. A prospective study in Spain showed that 25.1% of patients with schizophrenia spectrum disorders developed a predictor of NAFLD in 3 years [30]. Our result was lower than that in the Spain study, which might be related to the different NAFLD definitions between our study and the study in Spain. In our study, NAFLD was defined according to ICD-10 code. The study in Spain used fatty liver index to determine the presence of liver steatosis, which predicted fatty liver disease based on body mass index (BMI), waist circumference, and triglyceride and gamma-glutamyltransferase levels [30]. Yan et al. conducted a cross-sectional study in Liaoning, China, showing that the prevalence of NAFLD was 49.5% in 202 young males with schizophrenia [21]. In the present study, male schizophrenia inpatients aged 50–59 years had the highest NAFLD prevalence of 29.78%, which was still lower than that in the Liaoning study. The higher NAFLD prevalence in Liaoning study might be because that a large number of study population were more likely to use multiple antipsychotics combination [21].

In this study, results of multivariable logistic regression showed that compared to other mental disorders, schizophrenia and bipolar disorder were correlated with higher prevalence of NAFLD. These findings are in line with that of previous study showing schizophrenia and bipolar disorder were risk factors for NAFLD [18]. Patients with schizophrenia are closely related to chronic liver disease, due to higher prevalence of metabolic syndrome, unhealthy lives, and antipsychotics use [17]. In this study, results of multivariable analysis showed that inpatients who used antipsychotics were more likely to have NAFLD (aOR = 1.46, 95% CI 1.38–1.54). Results of subgroup analysis showed that regardless of mental disorder types, antipsychotics were correlated with higher prevalence of NAFLD, especially among inpatients with depressive disorder, which warrants more attention and further investigation. The use of antipsychotics could lead to NAFLD by causing weight gain, obesity, lipid metabolism alterations in mental disorder inpatients [13]. Meanwhile, there is concern about the hepatotoxicity potential for some antipsychotics, which may induce abnormalities in the liver biochemistry [31]. However, the adverse effect profiles varied from one antipsychotics to another, [31] and the antipsychotics differed between different mental disorder types. Further studies are needed to evaluate the risk of NAFLD for different type of antipsychotics.

We found that 17.89% of inpatients with bipolar disorder had NAFLD, which was lower than the prevalence of liver disease in veterans with bipolar disorder (21.5%) [18]. Results from the veterans study showed that the presence of bipolar disorder was a risk factor for NAFLD [18]. Our study also found that inpatients with bipolar disorder were more likely to have NAFLD than those with other mental disorder. In addition, we found that the prevalence of NAFLD peaked at 30–39 years for male inpatients with bipolar disorder. Given the young age at which NAFLD occurred in male bipolar disorder inpatients, these young males might have significant higher risk for developing NASH or cirrhosis over the course of their lives.

In the present study, the prevalence of NAFLD was 12.62% among inpatients with depressive disorder, which was lower than that among inpatients with schizophrenia and bipolar disorder. A study in US showed that the prevalence of NAFLD was 44.5–65.9% among people with depression according to different NAFLD definitions [19]. Our result was lower than the US study, which might be related to different study population and different NAFLD definition. Meanwhile, we found the prevalence of NAFLD was higher in young male depressive patients. This finding suggests that early screening and detection for NAFLD are needed in these inpatients. In our study, NAFLD was diagnosed by ultrasound, which was a simple, non-invasive diagnostic test, and the most commonly used imaging modality in the diagnosis of NAFLD [32]. However, ultrasound is less reliable for the detection of mild steatosis and stage of fibrosis [32]. Previous studies showed that diffusion-weighted magnetic resonance imaging (DW-MRI) is one of the promising techniques that might enhance the diagnostic accuracy of hepatic fibrosis [33]. The apparent diffusion coefficient (ADC) values can be calculated using DW-MRI, which can be used as a non-invasive method for detection and grading of hepatic fibrosis in children with chronic hepatitis.[34]. Thus, the ADC might have potential clinical application values in the diagnosis of stage of hepatic fibrosis among mental disorder patients.

In our study, we found that the prevalence of NAFLD was associated with comorbidities. Results from multivariable analysis showed that mental disorder in-patients with hypertension, diabetes, dyslipidemia had higher NAFLD prevalence than inpatients without these comorbidities, regardless of mental disorder types. The prevalence of NAFLD was especially high when schizophrenia comorbid with diabetes (41.33%). Our result was consistent with the previous studies, which showed that diabetes and other metabolic dysfunction were risk factors of NAFLD [11, 12]. The association of NAFLD with metabolic syndrome was mutual and bi-directional [12]. Recent study demonstrated that NAFLD was an early predictor of metabolic dysfunction, even in healthy populations without metabolic dysfunction [35]. Furthermore, presence of diabetes could accelerate the course of NAFLD and was a predictor of advanced fibrosis and mortality [11]. Thus, mental disorder inpatients especially those with components of metabolic syndrome should be screened and managed for NAFLD and comorbidities. Metabolic associated fatty liver disease (MAFLD), a new definition of fatty liver disease, was considered more practical and more capable of identifying at-high-risk patients than the previous NAFLD criteria [36]. The diagnosis of MAFLD is based on the detection of liver steatosis together with the presence of at least one of three criteria that includes overweight or obesity, type 2 diabetes or clinical evidence of metabolic dysfunctions [36]. However, MAFLD diagnosis could not be made in our study due to lack of information on BMI. Future studies are needed to investigate the prevalence of MAFLD among mental disorder inpatients.

To our knowledge, this study was the largest observational study of NAFLD in mental disorder inpatients in China, which covered all adult mental disorder inpatients from 19 specialized psychiatric hospitals in Beijing in 2014–2018. Our results provided the updated prevalence of NAFLD among Chinese mental disorder inpatients. These latest findings have strong implications for prevention and control of NAFLD in mental disorder inpatients. Second, we used ICD-10 codes for mental disorder, NAFLD, and comorbidities definition. The 19 hospitals included in our study were all second or third-level hospitals with better medical resources and experienced physicians. Experienced physicians diagnosed inpatients admitted in these hospitals according to guidelines. All diagnosis was coded according to ICD-10. Thus, the quality of data and study results are more reliable because of the accurate diagnosis.

This study also had several limitations. First, participants in our study were all inpatients from specialized psychiatric hospitals. Mental disorder patients in general hospitals and communities were not included. Thus, our results could not be generalized to the general mental disorder population. Second, information of some important risk factors of NAFLD was not available in our study, such as obesity, smoking, and alcohol consumption. Thus, these factors were not included in the analysis, which might affect the results of factors associated with NAFLD in our study. Third, information of the type of antipsychotics, other medications that could induce NAFLD and poly-pharmacy was not available. Thus, we could not analyze the association between different antipsychotics or other medications and NAFLD.

## Conclusion

NAFLD was common among Chinese mental disorder inpatients, and increased over years. The prevalence of NAFLD was higher among men, old women, inpatients with schizophrenia, bipolar disorder, inpatients used antipsychotics, and those comorbid with hypertension, diabetes, and dyslipidemia. Fatty liver disease among mental disorder population warrants the attention of psychiatric specialists and health policy-makers.

## Supplementary Information

Below is the link to the electronic supplementary material.

Supplementary file1 (DOCX 95 KB)

## Data Availability

Data may be obtained from a third party and are not publicly available.
