# Supplementary material for: Prevalence of nonalcoholic fatty liver disease in mental disorder inpatients in China: an observational study
Source: Hepatol Int. 2021 Jan 29;15(1):127–36. doi: 10.1007/s12072-020-10132-z (PMC7886739; doi:10.1007/s12072-020-10132-z)
Supplement: Supplementary file 1 — Supplementary file1 (DOCX 95 KB) [file 12072_2020_10132_MOESM1_ESM.docx]

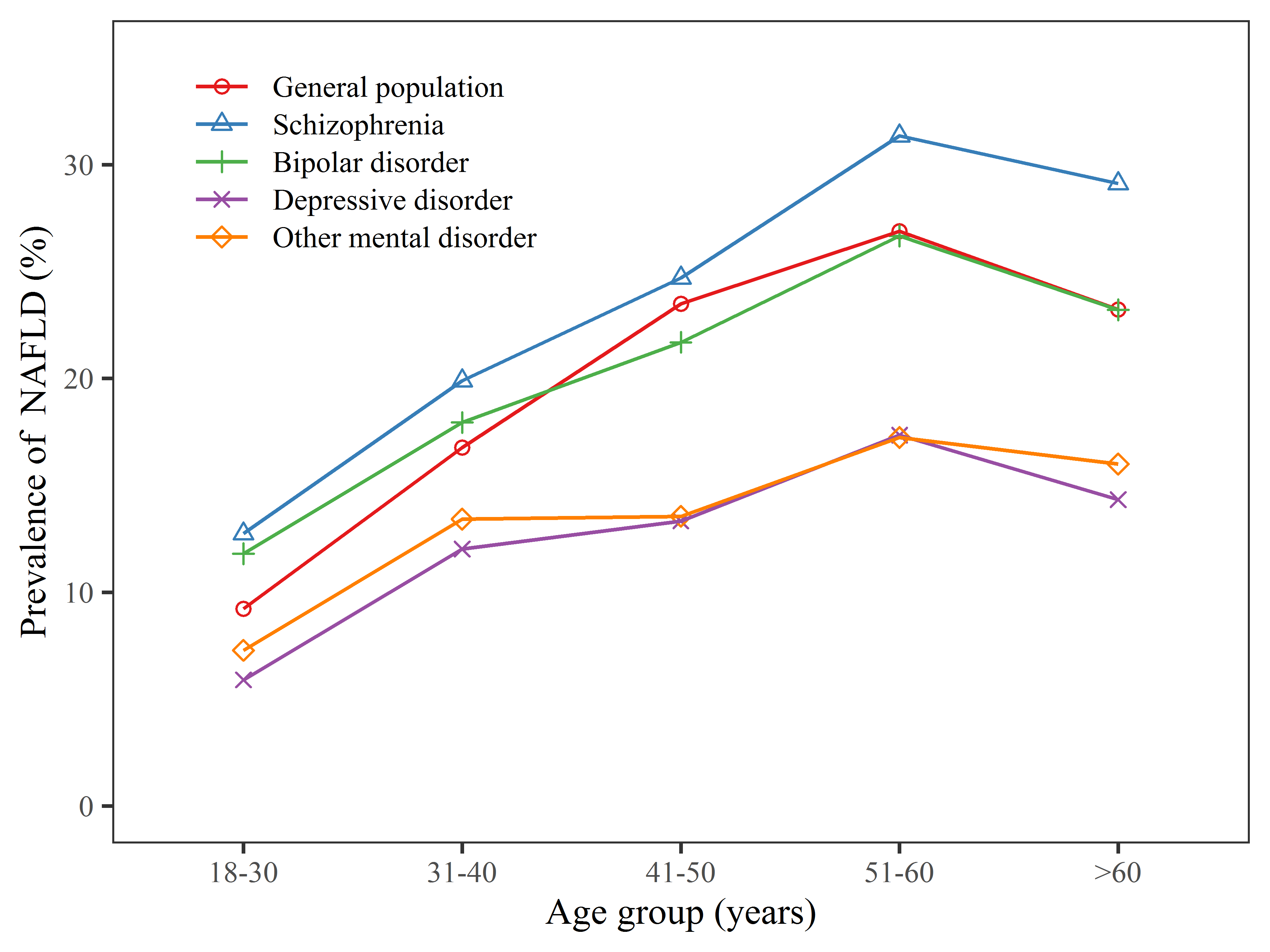


**Supplemental Figure 1. Age-specific prevalence of NAFLD among general population and mental disorder inpatients in China** The prevalence of NAFLD among general population in China (red line) was originally presented in a meta-analysis by Li et al. based on articles published from 1997 to 2013 [6].
